# Emerging and re-emerging vector-borne and other zoonotic RNA viruses: pathogenesis, climate-driven dynamics, and strategies for global control

**DOI:** 10.3389/fmicb.2026.1755594

**Published:** 2026-04-27

**Authors:** Niloofar Farsiu, Fatemeh Khodadadpour Mahani, Mohammad Rezaie Zadeh Rukerd, Nazanin Zeinali Nezhad, Mohammad Rahimi, Fatemeh Sadat Mirtajadini, Mohammad Pardeshenas, Mohsen Nakhaie, Pouya Hassandarvish

**Affiliations:** 1 Gastroenterology and Hepatology Research Center, Institute of Basic and Clinical Physiology Sciences, Kerman University of Medical Sciences, Kerman, Iran; 2 Research Center of Tropical and Infectious Diseases, Kerman University of Medical Sciences, Kerman, Iran; 3 Department of Stem Cells and Developmental Biology, Cell Science Research Center, Royan Institute for Stem Cell Biology and Technology, ACECR, Tehran, Iran; 4 Physiology Research Center, Institute of Neuropharmacology, Kerman University of Medical Sciences, Kerman, Iran; 5 Department of Microbiology, School of Medicine Kerman University of Medical Sciences, Kerman, Iran; 6 Social Determinants of Health Research Center, Institute for Futures Studies in Health, Kerman University of Medical Sciences, Kerman, Iran; 7 Clinical Research Development Unit, Afzalipour Hospital, Kerman University of Medical Sciences, Kerman, Iran; 8 Tropical Infectious Diseases Research & Education Centre, Universiti Malaya, Kuala Lumpur, Malaysia

**Keywords:** climate change, global health, pathogenesis, vector-borne viruses, zoonotic diseases

## Abstract

Vector-borne and other zoonotic RNA viruses provide a significant and growing threat to global health, especially in areas where climate change, urbanization, and population growth facilitate the proliferation of arthropod vectors. This review offers an extensive examination of the biology, epidemiology, and pathogenesis of numerous important viruses, including dengue, Zika, chikungunya, yellow fever, Japanese encephalitis, Crimean–Congo hemorrhagic fever, Nipah, Ebola, and hantaviruses. We underscore how environmental and social factors, particularly increasing temperatures, modified precipitation patterns, and accelerated urbanization, transform vector habitats and spillover dynamics. The article further analyzes host–virus and virus–vector interactions, highlighting mechanisms of immune evasion, neurotropism, and vascular disease. Computational and machine learning models are examined as novel instruments for forecasting outbreaks and developing early warning systems. Finally, a summary of present and prospective control options is provided, covering integrated vector management, Wolbachia-based biological control, vaccinations, and antiviral immunotherapies.

## 1 Introduction

Vector-borne and other zoonotic RNA viruses impose a substantial global public-health and economic burden, with disproportionate effects in low- and middle-income countries that are more vulnerable to outbreaks. Each year, hundreds of millions of people are infected with Aedes mosquito-borne viruses, including dengue virus (DENV), chikungunya virus (CHIKV), and Zika virus (ZIKV). For example, DENV accounted for >7.6 million new cases and >3,000 deaths by April 2024 and threatens nearly 100 million people worldwide (WHO, 2024). Aedes mosquito-borne diseases cost at least $94.7 billion globally between 1975 and 2020 (Roiz et al., 2024), and CHIKV incurred an additional estimated $47.1 billion from 2011 through 2020 (de Roo et al., 2024). Taken together, these viruses impose an estimated $15–20 billion in annual costs, including health services, lost productivity, tourism losses, and outbreak control efforts. Health-care systems bear immense pressure during outbreaks of DENV, Ebola virus (EBOV), and CHIKV, including intensive care unit (ICU) saturation, diagnostic overload, and diversion of resources from other priorities (WHO, 2025; Hamilton, 2017).

Significant health challenges are emerging due to climate variability and urban population expansion. The urban heat-island effect intensifies extreme heat and can interact with extreme precipitation, increasing exposure to health risks in densely populated cities (Li et al., 2021; Singh et al., 2020). The presence and transmission of mosquito-borne diseases in urban areas are closely linked to climate-change patterns: rising temperatures and erratic rainfall accelerate vector metabolism and expand breeding sites, elevating risks for diseases such as dengue (Permatasari and Masjud, 2024; El-Sayed and Kamel, 2020). In these settings, residents of informal settlements are at the highest risk; climate change also affects mental health, heightening insecurity and uncertainty, especially among people experiencing homelessness and climate displacement (Damte et al., 2023; Cianconi et al., 2023). Rapid urbanization in Cameroon, for instance, has increased the presence of *Anopheles gambiae* and *Aedes aegypti*, altering disease-transmission patterns (Alenou et al., 2023). As urban populations continue to grow, particularly in low- and middle-income countries, early-warning systems and platforms, paired with well-targeted response strategies, are necessary to prevent added public-health burden (Li et al., 2021). Although mitigation strategies for heat and pollution are being widely implemented, some interventions can inadvertently exacerbate other health risks, underscoring the value of multisector approaches that address multiple urban health challenges simultaneously (Ligsay et al., 2021).

To define the scope of this review, we focus on high-impact vector-borne and other zoonotic RNA viruses that span major lineages: flaviviruses [DENV, ZIKV, yellow fever virus (YFV), Japanese encephalitis (JEV) (Simmonds et al., 2017; de Zanotto and Gould et al., 2002; Lindenbach and Rice, 2003); an alphavirus (CHIKV) (Chen et al., 2018; Singh et al., 2019); Bunyavirales lineages including Phenuiviridae for Rift Valley fever (RVFV) (King et al., 2018; Gaudreault et al., 2019), Nairoviridae for Crimean-Congo hemorrhagic fever virus (CCHFV) (Garrison et al., 2020; Zivcec et al., 2016; Keşkek Türk and Kacı, 2024), and Hantaviridae for hantaviruses (Bradfute et al., 2024; Vial et al., 2023); a paramyxovirus (Nipah virus (NiV)) (Harcourt et al., 2000; Wang et al., 2001); and filoviruses (EBOV and Marburg virus (MARV)) (Biedenkopf et al., 2024)]. Despite their taxonomic diversity, these pathogens share programmatically relevant features, including small enveloped RNA genomes (positive or negative sense, segmented or non-segmented) and maintenance in animal reservoirs that enable zoonotic spillover. Transmission occurs either via arthropod vectors (primarily mosquitoes or ticks) or through direct or environmental exposure to infected animal hosts, and clinical syndromes range from self-limited febrile illness and congenital disease to encephalitis and hemorrhagic fever.

In this review, we synthesize current knowledge on host-virus interactions, pathogenesis, and transmission ecology, and we compare modeling and control strategies across taxa to identify common leverage points for surveillance, early warning, and targeted intervention.

## 2 Classification of viruses based on biological and epidemiological characteristics

**Figure 1:**
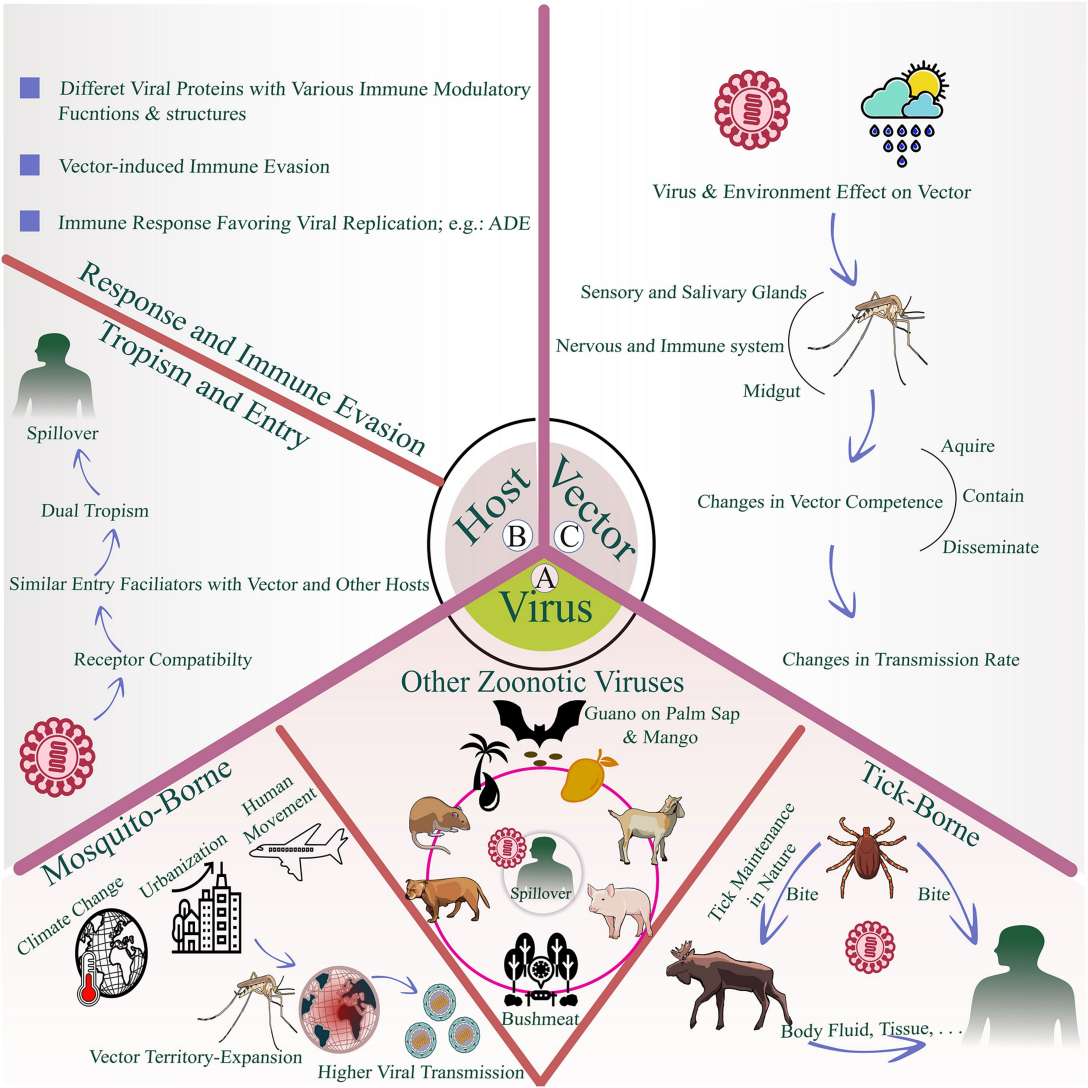
Schematic illustration of the intricate interactions between mosquito-borne, tick-borne, and other zoonotic RNA viruses with hosts and vectors: **(A)** Common routes by which viruses are maintained in nature and potential spillover pathways to human hosts; **(B)** Virus–host interactions that facilitate spillover, focusing on interactions with host cells and the immune system, including dual tropism, immune modulation, and immune responses; **(C)** Vector-related determinants of spillover and transmission, particularly for arboviruses, highlighting how changes in vector dynamics can influence viral transmission.

To classify the debated viruses, we use dominant transmission ecology as the primary organizing principle. Because many vector-borne viruses are also zoonotic (maintained in enzootic cycles with spillover to humans), our grouping is intended as a practical framework to compare pathogens that are principally shaped by arthropod-mediated transmission versus those acquired mainly through non-vector zoonotic exposure. For the vector-borne viruses, arthropod vectors are typically essential for efficient spillover and sustained transmission in nature; by contrast, other zoonotic viruses are acquired primarily through direct or indirect exposure to animal reservoirs (without an arthropod vector) (Taylor-Robinson, 2024). Importantly, vector-mediated transmission is not merely an epidemiological label; vector feeding introduces virus into the skin, along with salivary factors that can alter inoculation conditions (route, dose, timing), modulate early local immunity, influence dissemination kinetics, and, in some settings, augment disease severity (Visser et al., 2023; Schmid et al., 2016). All of these factors reshape both transmission and pathogenesis dynamics. Accordingly, we group representative viruses and summarize key epidemiological features. Figure 1A summarizes common routes by which viruses are maintained in nature and spill over to humans. The following subsection discusses these pathways in detail. Moreover, the key features of the viruses discussed in this review are summarized in Table 1.

**Table 1:** Comprehensive overview of major zoonotic and vector-borne RNA viruses discussed in this review, including their taxonomic classification, associated diseases, reservoir hosts, transmission routes, vectors, geographic distribution, and the current status of licensed vaccines and antiviral therapies.

Virus name	Family and genus	Associated disease	Primary reservoir hosts	Transmission route	Vector(s)	Geographic distribution	Licensed vaccines and antivirals	Key pathogenic/Epidemiological notes
Dengue virus (DENV)	Flaviviridae, Flavivirus	Dengue Fever, Dengue Hemorrhagic Fever, Dengue Shock Syndrome	Humans, primates, Aedes mosquitoes	Vector-borne, Aedes mosquito bite	*Aedes aegypti*, *Aedes albopictus*	Tropics, sub-tropics	Vaccine: Dengvaxia^®^ (dengue tetravalent vaccine)	Can cause severe disease, particularly in secondary infections due to antibody-dependent enhancement (ADE).
Zika virus (ZIKV)	Flaviviridae, Flavivirus	Zika Fever, Congenital Zika Syndrome	Humans, monkeys, Aedes mosquitoes	Vector-borne, Aedes mosquito bite	*Aedes aegypti*, *Aedes albopictus*	Tropics, sub-tropics	No licensed vaccine. Experimental vaccines under development.	Major cause of congenital microcephaly. Sexual transmission observed.
Chikungunya virus (CHIKV)	Togaviridae, Alphavirus	Chikungunya Fever	Non-human primates, birds	Vector-borne, Aedes mosquito bite	*Aedes aegypti*, *Aedes albopictus*	Africa, Asia, Europe, Americas	No licensed vaccine.	Associated with long-lasting joint pain, sometimes lasting years.
Yellow fever virus (YFV)	Flaviviridae, Flavivirus	Yellow Fever	Non-human primates, mosquitoes	Vector-borne, Aedes and Haemagogus mosquitoes	*Aedes aegypti*, Haemagogus spp.	Africa, South America	Vaccine: YF-VAX^®^ (live-attenuated 17D vaccine)	Disease can cause severe hepatitis, hemorrhagic fever, and organ failure.
Japanese encephalitis virus (JEV)	Flaviviridae, Flavivirus	Japanese Encephalitis	Pigs, waterbirds	Vector-borne, Culex mosquito bite	Culex tritaeniorhynchus	Asia, Pacific Islands	Vaccine: Inactivated (JE-VAX^®^), Live-attenuated (SA-14-14-2)	Major cause of viral encephalitis in Asia.
Crimean-Congo hemorrhagic fever virus (CCHFV)	Bunyaviridae, Nairovirus	Crimean-Congo Hemorrhagic Fever	Livestock (sheep, cattle), ticks	Vector-borne, tick bite or animal contact	Hyalomma spp.	Africa, Asia, Europe, Middle East	No licensed vaccine, experimental treatments with monoclonal antibodies under study.	High mortality rate, severe hemorrhagic manifestations.
Nipah virus (NiV)	Paramyxoviridae, Henipavirus	Nipah Virus Infection, Acute Encephalitis	Fruit bats (Pteropus spp.), pigs	Direct contact, airborne, bat-to-human, pig-to-human	None	South and Southeast Asia	No licensed vaccine, monoclonal antibody therapy under development.	Causes severe encephalitis and respiratory illness with high mortality.
Ebola virus (EBOV)	Filoviridae, Filovirus	Ebola Virus Disease	Fruit bats, non-human primates	Direct contact with infected body fluids	None	Sub-Saharan Africa	Vaccine: Ervebo^®^ (recombinant vesicular stomatitis virus-based vaccine)	High mortality, rapid progression, outbreaks often associated with bushmeat consumption.
Hantaviruses (various species)	Hantaviridae, Hantavirus	Hantavirus Pulmonary Syndrome (HPS), Hemorrhagic Fever with Renal Syndrome (HFRS)	Rodents (various species)	Inhalation of aerosolized rodent excreta	None	Worldwide	No licensed vaccine	Pulmonary edema, renal failure, high mortality in some strains.

### 2.1 Mosquito-borne viruses

Recognized mosquito-borne viruses, including DENV, ZIKV, YFV, and CHIKV, are raising substantial global concern and are expanding their geographic range. This expansion is driven largely by the widening distributions of *A. aegypti* and *Aedes albopictus* due to climate change, urbanization, and human movement (Kraemer et al., 2019; Messina et al., 2019). The basic reproduction number (R0) for these viruses varies by climate zone, with higher values in tropical and subtropical regions than in temperate areas (Liu et al., 2020). Recent estimates suggest that 5.66 billion people live in areas suitable for DENV, CHIKV, and ZIKV transmission, and that 1.54 billion individuals are at risk for YFV (Lim et al., 2025).

JEV shows distinct geographic patterns, shaped by genotype diversity (G1-G5), and is primarily distributed across Southeast Asia and the Western Pacific (Gao et al., 2019; Mulvey et al., 2021). Genotype 3 was historically dominant in Asia, but genotype 1 has become prevalent in recent years, while genotype 3 has also been detected in Europe and Africa (Gao et al., 2019).

RVFV is endemic in much of Africa and has expanded to the Middle East, northern Egypt, and the Comoros Archipelago (Mansfield et al., 2015). As with other mosquito-borne viruses, climate change and social disruption may further extend the ranges of JEV and RVFV (Mulvey et al., 2021; Mansfield et al., 2015).

Mosquito-borne viruses share several features, including common clinical manifestations such as fever and rash, similarities in protein structure, overlaps in tropism and immune evasion, and frequent serologic cross-reactions in laboratory assays (Cenci Dietrich et al., 2025). Clinical hallmarks nonetheless differ by pathogen: DENV is associated with hemorrhagic manifestations, ZIKV with congenital microcephaly, YFV with hepatitis, CHIKV with polyarthralgia, and JEV with acute encephalitis (Cenci Dietrich et al., 2025; Kyaw et al., 2019).

Transmission of DENV, ZIKV, YFV, JEV, CHIKV, and RVFV occurs primarily via competent Aedes and Culex mosquitoes (Manouana et al., 2024). *A. aegypti* is the dominant and most efficient vector for many arboviral infections, including DENV, ZIKV, YFV, and CHIKV (Powell, 2018). *A. albopictus* is also an efficient vector for DENV, ZIKV, and CHIKV and can outperform *A. aegypti* in some temperate settings (Soni et al., 2020; Gomard et al., 2020; Vega-Rua et al., 2013). Additional Aedes species with demonstrated or suspected competence include *Aedes hensilli*, *Aedes vexans*, *Aedes simpsoni*, *Aedes japonicus*, and *Aedes caspius* (Benelli and Romano, 2017; Faizah et al., 2020; de Wispelaere et al., 2017; Drouin et al., 2022; Turell et al., 2010). The Culex genus also contributes to arboviral transmission. Culex tritaeniorhynchus is the primary vector for JEV, while *Culex pipiens*, Culex annulirostris, and *Culex tarsalis* have roles in transmitting JEV and RVFV in specific regions (Faizah et al., 2020; de Wispelaere et al., 2017; Turell et al., 2010; Van den Eynde et al., 2022; Seufi and Galal, 2010).

Mosquito-borne viruses have been isolated from additional mosquito species, but vector competence remains to be fully evaluated in some locales. Transmission efficiency varies by virus strain and mosquito species. For example, YFV transmission by *A. aegypti* is typically highly efficient, yet in parts of Kenya, *A. simpsoni* has been reported to be even more efficient (Ellis et al., 2012). Consequently, local assessments of vector competence, seasonal vector density, host preference, and vector behavior are essential for accurate appraisal of mosquito-borne disease risk.

### 2.2 Tick-borne viruses

Over 35 virus species across five families are transmitted by ticks worldwide. Global circulation of these viruses has risen due to increased exposure to tick bites; tick populations are also expanding (Madison-Antenucci et al., 2020; Yu and Park, 2024). Among tick-borne viruses, CCHFV is among the most widespread, with detection across Africa, Europe, the Middle East, and Asia. Six genetic lineages based on S-segment sequence homology have been described, each associated with characteristic regions: Group I (West Africa), Group II (Democratic Republic of the Congo), Group III (South and West Africa), Group IV (Asia and the Middle East), Group V (Europe and Turkey), and Group VI (Greece) (Yu and Park, 2024; Carroll et al., 2010). The principal tick vector for CCHFV is *Hyalomma marginatum* (Ergönül, 2006). Hyalomma species serve as both vectors and reservoirs for CCHFV. Small mammals, ground-feeding birds, and large ungulates develop brief viremia without clinical disease; although they are not long-term reservoirs, these hosts are crucial for sustaining tick populations and maintaining CCHFV in nature (Madison-Antenucci et al., 2020; Gargili et al., 2017). Humans acquire infection via bites from infected ticks or through contact with blood or tissues of infected animals; human-to-human transmission can occur through exposure to body fluids or organs of infected individuals (Yu and Park, 2024; Bente et al., 2010). Clinical presentation ranges from nonspecific febrile illness to severe disease with mucosal or gastrointestinal bleeding; in severe cases, case fatality can be high (Spengler et al., 2016; Bente et al., 2013).

### 2.3 Other zoonotic RNA viruses

Having outlined vector-borne viruses, where arthropod transmission largely shapes exposure, early immune events, and outbreak dynamics, we now turn to zoonotic RNA viruses in which human infection is typically acquired through non-vector routes, such as contact with infected animals, exposure to contaminated secretions and excreta, or intermediate hosts. In this subsection, we discuss NiV, EBOV, MARV, and hantaviruses. Human NiV infections have been reported in South and Southeast Asia, including India, Bangladesh, Malaysia, Singapore, and the Philippines (Epstein et al., 2020). EBOV and MARV are endemic to central Africa but show different geographic patterns: EBOV is mainly found in humid rainforests of central and western Africa, whereas MARV is more often reported from drier areas of central and eastern Africa (MacNeil and Rollin, 2012; Peterson et al., 2004). Hantaviruses are endemic in Asia, Europe, and the Americas; clinical syndromes differ by region, with hantavirus cardiopulmonary syndrome (HCPS) predominant in the Americas and hemorrhagic fever with renal syndrome (HFRS) common in Eurasia (de Oliveira and Faccini-Martínez, 2020). NiV affects both the respiratory and nervous systems, causing acute respiratory distress and encephalitis (Bossart et al., 2009; Devnath et al., 2022). EBOV and MARV cause severe viral hemorrhagic fever with very high case fatality in some outbreaks (Olival and Hayman, 2014). Bats are considered the primary reservoirs for NiV, EBOV, and MARV, with fruit bats in the family Pteropodidae implicated as long-term hosts (Epstein et al., 2020; Wang and Anderson, 2019). For NiV, spillover has been linked to bat excreta contaminating raw date palm sap and to bat-contaminated fruit, with pigs serving as amplifying hosts in some outbreaks; direct bat-to-human transmission and pig-to-human transmission have both been documented (Altizer et al., 2018; de Wit and Munster, 2015). EBOV spillover has been associated with hunting, butchering, and consumption of bushmeat (Judson et al., 2016). Serologic and exposure studies suggest that dogs and small ruminants can be exposed to EBOV and MARV (Ndong Mebaley et al., 2025). Hantaviruses are typically maintained in rodent reservoirs, and each virus type is usually associated with a specific rodent species. Some species have been detected in shrews, moles, and bats (Forbes et al., 2018). People typically get infected by breathing in tiny particles from rodent urine, droppings, or saliva, especially in areas with lots of rodents (Mittler et al., 2019) (Figure 1A).

## 3 Virus-host and virus-vector interactions

### 3.1 Host tropism and entry mechanisms

Viral infection begins with receptor recognition, a molecular interaction that dictates tropism and shapes disease trajectory. The presence of suitable entry receptors (susceptibility) does not by itself ensure a productive replication cycle (permissiveness) (Manjarrez-Zavala et al., 2013). Therefore, tropism should be examined both at the level of entry and with respect to the host cell’s capacity to support replication. For vector-borne and other zoonotic RNA viruses, dual tropism and conserved receptor usage are key features that help these pathogens overcome cross-species transmission barriers. In EBOV infection, multiple cell-surface attachment factors facilitate entry, including T-cell immunoglobulin and mucin domain 1 (TIM-1), dendritic cell-specific ICAM-3-grabbing non-integrin (DC-SIGN), and members of the TAM receptor family (Tyro3, Axl, Mer). Niemann–Pick C1 (NPC1) functions as the essential endosomal receptor, and recent work reports that Sudan virus (SUDV) binds NPC1 with higher affinity than some strains, potentially enabling infection of cells with lower NPC1 expression and contributing to broader cell tropism (Bu et al., 2025). Following entry, EBOV preferentially infects monocytes and dendritic cells that traffic to lymphoid tissues and disseminate infection systemically; subsequent spread to hepatocytes, adrenal cortical cells, and vascular endothelial cells leads to multiorgan involvement, particularly in the liver and spleen (Jankeel et al., 2020; Letafati et al., 2023). For flaviviruses such as ZIKV and DENV, overlapping receptor usage in humans has been documented for DC-SIGN, TIM-1, and the TAM receptors Tyro3, Axl, and MerTK. Despite these shared entry factors, ZIKV and DENV differ markedly in tropism. ZIKV shows pronounced neurotropism, a feature rarely observed with DENV, a divergence that may reflect differences in systemic dissemination and cell-intrinsic replication programs. Neuroinvasive viruses such as ZIKV and NiV can exploit a “Trojan horse” mechanism, using infected monocytes or macrophages to traverse the blood–brain barrier and the placental barrier (Zhang et al., 2023; Al-Obaidi et al., 2024; de Carvalho et al., 2019). NiV, a zoonotic paramyxovirus, exemplifies broad tropism through its use of Ephrin-B2 and Ephrin-B3 receptors. These receptors are conserved across species and are expressed on endothelial cells and neurons, which helps explain NiV’s propensity to cause systemic vasculitis and encephalitis (Navaratnarajah et al., 2020; Xu et al., 2012).

### 3.2 Immune evasion and host responses

After entry into a permissive host cell, viral replication remains constrained by host factors, and multiple mechanisms can inhibit genome amplification and progeny production. The interaction between viruses and the host immune system is complex and dynamic.

#### 3.2.1 Virus-intrinsic immune evasion mechanisms

While the interactions between viruses and the host immune system can lead to detection and elimination of the virus, many viruses have evolved strategies to evade or delay immune recognition, thereby gaining a foothold before the host can respond effectively. Structural and molecular differences among viral proteins can result in marked variation in the modulation of immune responses. A useful example is the comparison of two pathogenically important zoonotic filoviruses. While EBOV and MARV both encode proteins with similar names and functions, the ability of these proteins to inhibit immune responses varies. These viruses each encode a VP35 protein that suppresses RIG-I-like receptor signaling and interferon (IFN)-*α*/*β* production by several mechanisms, including direct binding to double-stranded RNA (dsRNA). However, one study showed that in cell culture, MARV infection induces greater upregulation of IFN responses than EBOV infection. This correlates with differences in the efficiencies by which EBOV and MARV viral protein 35 (VP35) antagonize RIG-I signaling. Previous studies have shown that MARV VP35 is a trimer in contrast to EBOV VP35, and this oligomeric change may contribute to the differences (Xu et al., 2012; Bruhn et al., 2017). MARV and EBOV also encode the VP24 protein. While this protein supports immune evasion in both viruses, unlike VP35, it does not exert the same functionality. EBOV VP24 is a strong immunosuppressive protein that blocks IFN signaling by preventing the nuclear import of phosphorylated signal transducer and activator of transcription 1 (STAT-1), a key step in the host antiviral response. In contrast, MARV VP24 induces cytoprotective antioxidant responses through an interaction with the host protein Keap1, which prevents apoptosis, promotes cell survival, and supports viral replication (Vogel et al., 2023).

#### 3.2.2 Vector-mediated immune modulation

There are also other ways viruses can escape immune recognition that are less discussed. One illustrative immune-evasion mechanism used by vector-borne viruses derives from their transmitting vectors. It has been shown that, for instance, *Hyalomma anatolicum* ticks transmitting CCHFV can modulate host complement pathways to evade immediate immune responses, facilitating prolonged feeding. This vector-driven immune modulation also favors viral transmission to the host, enhancing evasion from innate immunity (Mood et al., 2024).

#### 3.2.3 Host immune responses and immunopathology

Immune-evasion strategies are not always fully successful, and the host immune system can still mount effective antiviral responses. In the case of hantaviruses, a study showed that although the virus upregulates the immune checkpoint ligands programmed-death ligand 1 (PD-L1) and PD-L2, which typically suppress T-cell activity, this does not entirely prevent immune activation. Remarkably, hantavirus infection still elicits robust bystander activation of CD8 + T cells, which appears to bypass the inhibitory PD-1 pathway. This suggests that despite the virus’s attempts to dampen immune responses, the host can partially overcome these strategies, allowing early antiviral defenses that may contribute to disease resolution in many cases (Raftery et al., 2018). An important issue in dealing with infectious diseases is that immune activation is not always protective; paradoxically, it can also be the cause of disease. In many viral infections, it is the immune system’s overreaction, rather than the virus itself, that leads to tissue damage, organ failure, and severe clinical outcomes. Regarding zoonotic RNA viruses, it has been observed that the host responses can change the outcome. For example, many zoonotic viruses do not cause disease in their natural reservoir but lead to severe outcomes in human cases. While this can be due to the inability of the host immune system to limit viral infection and replication, in cases such as ZIKV-associated damage of the central nervous system, excessive and imbalanced inflammatory responses have been implicated in more severe pathology. In one study, neonates with microcephaly due to congenital ZIKV infection showed a substantially higher degree of inflammatory perturbation, with uncoupled inflammatory responses and reduced correlations among inflammatory biomarkers compared to those with the same infection but without microcephaly (Nascimento-Carvalho et al., 2021). In this context, antibody-dependent enhancement (ADE) is a critical consideration, particularly in flavivirus infections like DENV. While cross-reactive antibodies can sometimes protect, they may also worsen disease by opsonizing virions for uptake via Fc receptors on immune cells, especially during secondary infections with a different serotype. Although ADE has not been definitively confirmed in ZIKV, evidence suggests that preexisting DENV antibodies could modulate ZIKV disease outcomes, raising important considerations for co-circulating regions and for vaccine development (Maucourant et al., 2019).

Figure 1B illustrates a second key determinant of viral spillover to humans; interactions between the virus and host cells and the immune system. It highlights dual tropism, immune evasion, and immune responses, as discussed in Sections 3.1 and 3.2.

### 3.3 Vector competence and transmission dynamics

As outlined earlier, viral entry into the host can reshape host physiology and immunity. However, the impact of vector-borne viruses extends beyond host cells to their arthropod vectors, which they can manipulate to enhance transmission. Because these viruses depend on vectors to reach vertebrate hosts, it is essential to understand vector competence, defined as the intrinsic capacity of a vector to acquire, sustain, and transmit a virus. Despite its importance, experimental investigations of mosquito vector competence parameters remain limited. Certain viruses actively alter vector behavior to improve transmission. For DENV, infection disrupts key sensory pathways and salivary gland function in Aedes mosquitoes, increasing attraction to human hosts while reducing feeding efficiency; infected females thus take multiple short blood meals, which increases transmission opportunities (Wei Xiang et al., 2022).

ZIKV similarly alters neurotransmitter levels in *A. aegypti*, modifying feeding behavior (Gao et al., 2025). By contrast, West Nile virus (WNV) infection in *Culex pipiens* has been associated with reduced host-seeking, a change not explained by antennal responsiveness and therefore likely reflecting disruption of central neural circuits (Vogels et al., 2017). These phenotypes arise through mutation and selection that favor variants with greater transmission success, rather than by deliberate viral strategy. The virus–vector interplay is bidirectional. Within the mosquito, viruses encounter physical, cellular, microbial, and immunological barriers shaped by vector genetics and local environmental conditions. These pressures drive viral evolution and influence transmission dynamics (Ketkar et al., 2019). For example, Chen et al. (2023) compared geographically distinct *Aedes aegypti* populations and found differences in immune-gene expression and microbiota composition that correlated with variable susceptibility to DENV-2, suggesting that local adaptation of immune pathways and symbioses modulates vector competence and transmission potential. At the molecular level, changes in salivary gland proteins, midgut receptors, and antiviral pathways can either facilitate or restrict viral replication and dissemination. Such adaptations are molded by environmental selection and ongoing viral circulation, yielding more efficient transmission phenotypes (Carpenter and Clem, 2023). Disruption of the RNA interference (RNAi) pathway illustrates this principle: knockout of Dicer-2 in *Aedes aegypti* increases replication and spread of CHIKV, Mayaro virus, DENV, and ZIKV, although CHIKV-infected mutants show reduced survival, indicating a fitness trade-off (Merkling et al., 2023). Thus, while weakening antiviral defenses can enhance transmission under some conditions, other mutations may reduce vector competence or fitness, highlighting the complexity of host-virus-vector evolutionary dynamics.

Figure 1C highlights an additional determinant of spillover and onward transmission, especially for arboviruses, showing how changes in vector dynamics can influence viral transmission.

## 4 Pathogenesis of high-consequence viruses

To enable clearer comparisons across the many viral families, we have organized this section around the dominant pathogenic strategies rather than following a strict virus-by-virus format (Begeman et al., 2023). Although these pathogens differ markedly in taxonomy and ecology, they often converge on similar biological pathways that dictate disease severity in humans. Framing pathogenesis in terms of major processes, therefore, highlights both shared mechanisms and critical distinctions among the agents. Accordingly, the discussion is divided into three principal patterns: (1) neuro-invasion and neuroinflammatory injury, (2) vascular dysfunction leading to hemorrhagic disease, and (3) pulmonary or renal tropism associated with systemic organ failure (De Vries and Harding, 2023). This structure allows readers to appreciate how disparate viruses can produce comparable clinical outcomes through common mechanistic routes, while also recognizing the unique features that set each pafthogen apart.

### 4.1 Neuro-invasion and neuroinflammatory injury

A concerning trend is the emergence of RNA viruses capable of invading the central nervous system (CNS) and triggering neuroinflammation (De Vries and Harding, 2023). Despite belonging to different viral families, viruses like ZIKV, JEV, NiV, and CHIKV share similar disease-causing pathways (De Vries and Harding, 2023; Al-Obaidi et al., 2024). These involve weakening the blood–brain barrier (BBB), directly infecting neurons, and triggering an immune response that damages the nervous system. This can lead to a range of neurological problems, from developmental issues in infants to acute encephalitis and lasting cognitive difficulties (Srichawla et al., 2024).

Neurotropic viruses represent a growing threat to global neurological health, causing complications that range from developmental anomalies to acute encephalitis and long-term cognitive deficits. Among these, ZIKV, JEV, NiV, and CHIKV have exerted a particularly profound impact on the central nervous system through varied but overlapping pathogenic mechanisms. ZIKV is transmitted by Aedes mosquitoes and primarily targets neural progenitor cells after peripheral infection, with entry facilitated by receptors such as Axl and DC-SIGN (Weaver et al., 2016; Pool et al., 2019; Hamel et al., 2015). Following vertical transmission, ZIKV can cross the placental barrier and establish infection within the developing fetal brain (Merfeld et al., 2017). This disrupts neurogenesis and can lead to congenital Zika syndrome, characterized by microcephaly, cortical thinning, ventriculomegaly, and ocular defects (Scotto et al., 2023). Even in infants without apparent birth defects, prenatal exposure can impair neurodevelopment later in life (Einspieler et al., 2019). In adults, ZIKV infection has been associated with Guillain–Barré syndrome (GBS), meningoencephalitis, and prolonged neuropathic pain (da Silva et al., 2017). These outcomes reflect a combination of direct neuro-invasion and immune-mediated injury, with evidence for a para-infectious component in GBS (Parra et al., 2016; Sánchez et al., 2021).

JEV, transmitted by Culex mosquitoes, can breach the blood–brain barrier through cytokine-driven inflammation or direct endothelial infection (Chen et al., 2014; Chang et al., 2015). Once inside the CNS, JEV displays tropism for neurons in the thalamus, hippocampus, and brainstem, leading to neuronal apoptosis, microglial activation, and extensive neuroinflammation (Johnson et al., 1985). Clinical manifestations include acute encephalitis, seizures, flaccid paralysis, and Parkinsonian features (Cody et al., 2024; Misra and Kalita, 2010; Solomon et al., 1998; Misra and Kalita, 1997). A substantial proportion of survivors experience persistent neurological sequelae such as cognitive impairment, psychiatric symptoms, and motor dysfunction, underscoring the depth and irreversibility of tissue injury (Campbell et al., 2011; Turtle et al., 2019; Yin et al., 2015).

**Figure 2:**
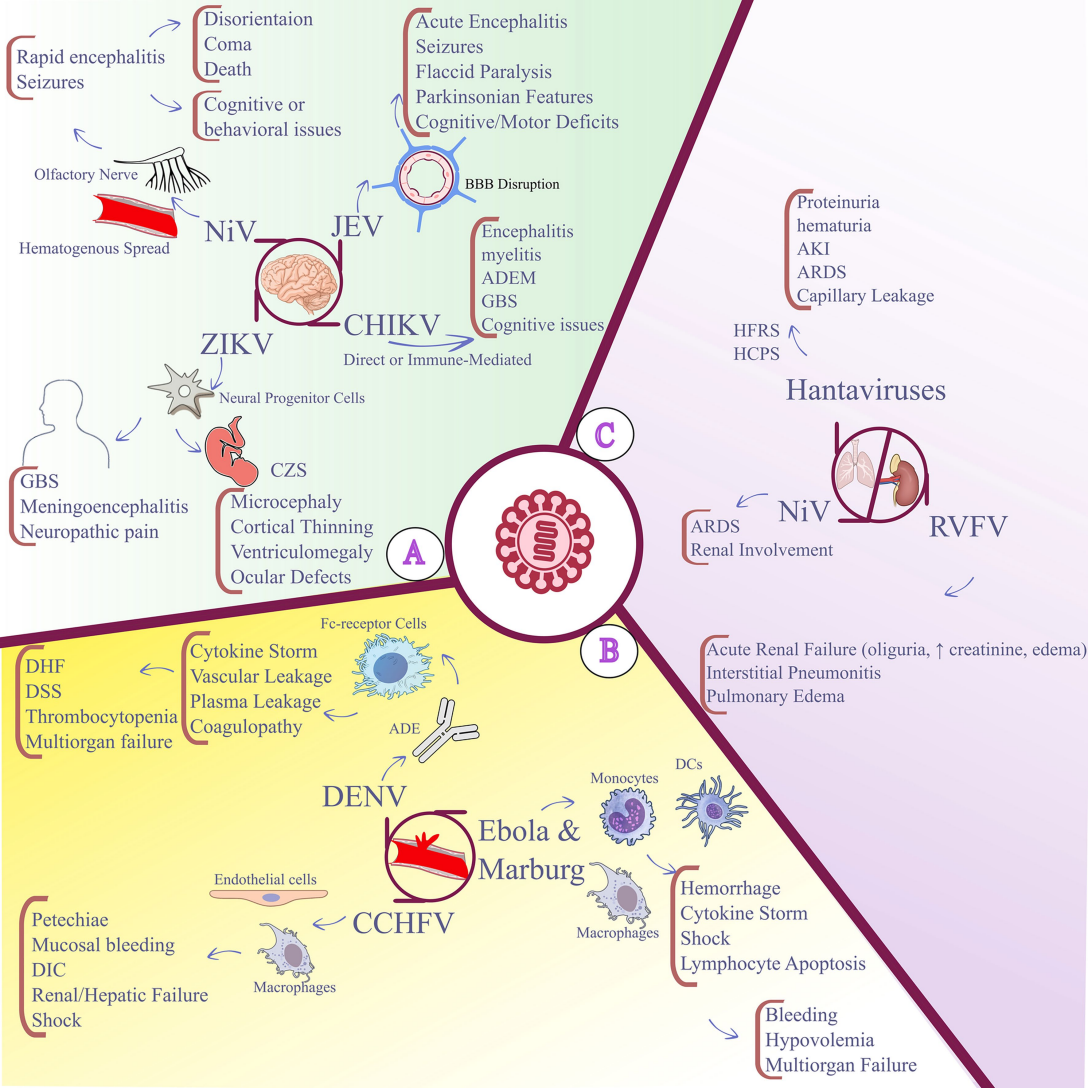
Overview of neurotropic, respiratory, hepatic, renal, and vascular pathogenesis and associated clinical outcomes in vector-borne and other zoonotic viral infections. **(A)** Neurologic infection and outcomes associated with ZIKV, NiV, JEV, and CHIKV. **(B)** Hemorrhagic and vascular pathogenicity of DENV, CCHFV, Ebola, and Marburg viruses. **(C)** Respiratory, renal, and hepatic involvement in NiV, hantaviruses, and RVFV. ZIKV, Zika virus; NiV, Nipah virus; JEV, Japanese encephalitis virus; CHIKV, chikungunya virus; DENV, dengue virus; CCHFV, Crimean–Congo hemorrhagic fever virus; RVFV, Rift Valley fever virus; CNS, central nervous system; CZS, congenital Zika syndrome; GBS, Guillain-Barré syndrome; DHF, dengue hemorrhagic fever; DSS, dengue shock syndrome; DIC, disseminated intravascular coagulation; ARDS, acute respiratory distress syndrome; AKI, acute kidney injury; HFRS, hemorrhagic fever with renal syndrome; HCPS, hantavirus cardiopulmonary syndrome.

NiV causes rapidly progressive encephalitis with a high case fatality rate (Siva et al., 2009; Bradel-Tretheway et al., 2019). The virus reaches the CNS via hematogenous routes that produce endothelial infection, vasculitis, and blood–brain barrier compromise, and it may also ascend through the olfactory nerve to access brain tissue directly (Altizer et al., 2018; Epstein et al., 2006; Talukdar et al., 2023). NiV infection produces widespread neuronal necrosis, microhemorrhages, and vasculopathy (Talukdar et al., 2023). Clinically, patients may develop disorientation, seizures, and coma, with death occurring within days of symptom onset (Siva et al., 2009; Singh et al., 2019). Survivors frequently have chronic neurological sequelae, including seizure disorders, personality change, and cognitive dysfunction (Bradel-Tretheway et al., 2019). Although CHIKV is best known for arthralgia and arthritis, neurological complications are increasingly recognized (Noret et al., 2012). The virus can affect the CNS through direct neuro-invasion and immune-mediated mechanisms (Puccioni-Sohler et al., 2023). Reported manifestations include encephalitis, myelitis, acute disseminated encephalomyelitis, and GBS (Cerny et al., 2017; Mehta et al., 2018; Musthafa et al., 2008; Cao-Lormeau et al., 2016). Long-term cognitive impairment, particularly among older adults, has also been observed (Peixoto et al., 2022). The pathophysiology likely combines viral replication in neural tissues with aberrant immune responses such as molecular mimicry and immune-complex deposition that promote demyelination and neuronal damage (Mehta et al., 2018; Goh et al., 2014). In conclusion, ZIKV, JEV, NiV, and CHIKV demonstrate how emerging and re-emerging viruses breach CNS defenses and initiate complex neuropathology. Elucidating their neuro-invasion routes and injury mechanisms is essential for improved diagnostics, prevention, and targeted therapies. Figure 2A summarizes neurologic infection and associated clinical outcomes for the viruses discussed above.

### 4.2 Vascular dysfunction and hemorrhagic pathogenesis

Another significant way these zoonotic RNA viruses cause illness is by damaging blood vessels and disrupting the body’s natural clotting coagulation processes. Viruses like DENV, CCHFV, EBOV, and MARV may have different underlying biology, but they all can harm endothelial barriers that line human blood vessels, trigger an overactive inflammatory response, and interfere with blood clotting (Muhsin et al., 2025). This cascade of events can result in fluid leaking from blood vessels, severe bleeding, and widespread organ failure in serious infections (Batista et al., 2025).

Hemorrhagic viruses pose major public-health risks because they can produce severe systemic disease characterized by plasma leakage, coagulation disorders, and multiorgan failure. DENV, CCHFV, EBOV, and MARV each disrupt vascular integrity through distinct mechanisms that converge on life-threatening complications. DENV, a member of the Flaviviridae family (Yousaf et al., 2018), primarily affects the vascular and immune systems (Zerfu et al., 2023). Primary infections are often mild or asymptomatic (Khan et al., 2023), but severe disease is more likely during secondary infection with a heterologous serotype (Guzman et al., 2013). ADE allows non-neutralizing antibodies to facilitate Fc-receptor-mediated uptake of virions into immune cells, increasing replication and cytokine production. Resultant cytokine surges promote vascular permeability, plasma leakage, and coagulopathy (Khan et al., 2023). Severe manifestations include dengue hemorrhagic fever and dengue shock syndrome, with hypovolemic shock, thrombocytopenia, and multiorgan dysfunction (Dash et al., 2006). Endothelial infection, platelet dysfunction, and dysregulated immunity drive vascular fragility and impaired hemostasis (Zerfu et al., 2023; Nachman and Rafii, 2008). Notably, severe dengue often peaks after viremia declines, highlighting the central role of host responses (Zerfu et al., 2023; Green and Rothman, 2006).

CCHFV, transmitted by ticks or contact with infected tissues, produces illness ranging from mild fever to fulminant hemorrhagic fever (Frank et al., 2024). After initial infection of endothelial cells and macrophages, the virus disseminates systemically, causing direct cytopathic injury and marked vascular damage (Bente et al., 2013; Gul et al., 2012; Oygar et al., 2023). Upregulation of endothelial adhesion molecules and release of proinflammatory cytokines exacerbate permeability and activate coagulation (Oygar et al., 2023; Akıncı et al., 2013). Patients can develop petechiae, mucosal bleeding, disseminated intravascular coagulation, hepatic dysfunction, renal impairment, and refractory shock; neurological signs such as confusion and agitation may occur during the hemorrhagic phase (Bente et al., 2013; Flick and Whitehouse, 2005; Cevik et al., 2008; Swanepoel et al., 1989). Mortality commonly results from multiorgan failure compounded by profound coagulopathy (Swanepoel et al., 1989; Bakir et al., 2022).

EBOV exhibits rapid and destructive pathogenesis following mucosal or parenteral entry (Feldmann and Geisbert, 2011). Early targeting of monocytes, macrophages, and dendritic cells permits dissemination through lymphatic and vascular routes (Geisbert et al., 2003a; Schnittler and Feldmann, 1998). Replication in endothelial cells, liver, and adrenal glands disrupts barrier function and coagulation (Feldmann and Geisbert, 2011). Hemorrhagic signs, including petechiae, mucosal bleeding, and visceral hemorrhage, arise from endothelial damage and dysregulated coagulation pathways, with tissue factor expression by infected monocytes as a key mechanism (Feldmann and Geisbert, 2011; Geisbert et al., 2003b). Fatal cases are marked by uncontrolled inflammation with excessive cytokine production that precipitates shock, metabolic derangements, and multiorgan failure (Turell et al., 2010). Lymphocyte apoptosis, influenced by host mediators such as nitric oxide and by viral proteins, contributes to immune collapse (Iampietro et al., 2017).

MARV follows a similar trajectory. Initial infection of macrophages and dendritic cells is followed by extensive replication in lymphoid tissues, liver, and adrenal glands (Alves et al., 2010). Hemorrhagic manifestations, including mucosal bleeding, petechiae, and internal hemorrhage, are frequent in the early organ phase (Brauburger et al., 2012). With progression, vascular permeability increases and leads to hypovolemia, shock, and multiorgan dysfunction (Feldmann and Geisbert, 2011; Brauburger et al., 2012). Dysregulated immunity, including exuberant cytokine release and bystander lymphocyte apoptosis, amplifies tissue injury (Warfield et al., 2007; Geisbert et al., 2000). Coagulopathy, often driven by liver damage and inflammatory mediators, is a major cause of death (Brauburger et al., 2012; Hensley et al., 2011). In summary, hemorrhagic viruses compromise vascular and immune homeostasis through combined cytopathic injury, immune hyperactivation, and coagulopathy, producing life-threatening disease that requires urgent supportive care and, where available, pathogen-specific interventions. Figure 2B summarizes key pathogenicity patterns in hemorrhagic and vascular viral infections.

### 4.3 Pulmonary, renal, and hepatic tropism with systemic organ failure

A third common disease pattern involves viruses that primarily target the lungs and kidneys, ultimately leading to widespread organ failure. Viruses like NiV, hantaviruses, and RVFV demonstrate how infection of the blood vessel lining, leakage from capillaries due to the immune system, and a virus’s preference for specific organs can combine to cause severe problems in the respiratory, kidney, and liver systems.

Emerging and re-emerging zoonotic RNA pathogens such as NiV, hantaviruses, and RVFV are geographically expansive and clinically severe because of their tropism for the pulmonary and renal systems. These infections illustrate how immune dysregulation, endothelial injury, and targeted tropism culminate in systemic organ failure. NiV is neurotropic and pneumotropic, entering primarily through the respiratory tract (Pandey et al., 2024; Hossain et al., 2008). Early in infection, viral antigens are detected in bronchiolar epithelium, type II pneumocytes, and alveoli, and are associated with cytokines such as interleukin (IL)-1β, IL-6, IL-8, and granulocyte-colony stimulating factor (Rockx et al., 2011; Escaffre et al., 2013). These mediators help drive acute respiratory distress syndrome (Rockx et al., 2011). As infection progresses hematogenously and via the olfactory pathway, the virus compromises the blood–brain barrier and disseminates to multiple organs, including the kidneys (Pandey et al., 2024; Rockx et al., 2011). Cough, dyspnea, and atypical pneumonia are common, particularly in outbreaks in Bangladesh and India (Hossain et al., 2008; Skowron et al., 2021). Renal involvement may arise secondarily through systemic vasculitis, endothelial infection, and multiorgan failure (Singh et al., 2019).

Hantaviruses target the cells that line our blood vessels. Surprisingly, they do not directly damage these cells; instead, the illness is mainly caused by the body’s immune system reacting and disrupting the function of these cells. This leads to leaky blood vessels and capillaries, which explains the two main illnesses people get: HFRS in Eurasia and HCPS in the Americas. Right now, the best way to prevent hantavirus infections is to limit contact with rodents by keeping environments clean, controlling rodent populations, and educating the public, as there is no widely available vaccine (Avšič-Županc et al., 2019; Schönrich et al., 2015). In HFRS, renal disease predominates, with proteinuria, hematuria, and acute kidney injury (Mir, 2022). The virus infects renal endothelial cells, podocytes, and tubular epithelium, disrupting tight junctions and raising vascular permeability (Krautkrämer et al., 2011). Despite significant proteinuria and interstitial edema, direct cytopathic damage is usually limited, indicating an immunopathological basis driven by cytokines and T-cell responses (Mir, 2022). HCPS features profound pulmonary edema from capillary leakage in the lungs (Koehler et al., 2022). Elevated vascular endothelial growth factor and bradykinin contribute to endothelial dysfunction, and CD8 + T-cell inflammation further increases permeability (Avšič-Županc et al., 2019; Marsac et al., 2011). Acute respiratory distress in HCPS is rapid and often fatal, with frequent need for mechanical ventilation (Duchin et al., 1994). Renal impairment, including proteinuria and acute kidney injury, also contributes to mortality in severe HCPS (Clement et al., 2014).

RVFV shows broad tissue tropism, with prominent hepatic and renal involvement (Adam et al., 2010). Renal manifestations include acute renal failure with oliguria, elevated creatinine, and systemic fluid overload such as pedal or pulmonary edema. These complications reflect combined direct cytopathic effects, hypovolemia from vascular leakage, and multiorgan dysfunction (El Imam et al., 2009). Pulmonary involvement, although less common, includes cough, dyspnea, and occasional viral pneumonia (Anywaine et al., 2022). Histopathology often shows interstitial pneumonitis and pulmonary edema. RVFV induces strong inflammatory responses that include interleukin-1β and tumor necrosis factor alpha, which may exacerbate vascular permeability and worsen both renal and pulmonary injury (de St Maurice et al., 2018; Linderholm et al., 1996).

Collectively, these pathogenic patterns highlight a significant observation that, despite considerable taxonomic differences, emerging zoonotic RNA viruses frequently converge on a relatively small number of disruptions to host pathways. These shared mechanisms, such as neuroinflammation, endothelial injury, and immune-mediated organ damage, offer a valuable framework for understanding the severity of disease and for identifying promising targets for future therapeutic development. Figure 2C illustrates respiratory, hepatic, and renal pathogenicity associated with hantaviruses, NiV, and RVFV.

## 5 Zoonotic spillover and emerging threats

### 5.1 Animal reservoirs and spillover risks

Zoonotic spillover events are pivotal in the emergence of novel human pathogens, with approximately 75% of emerging infectious diseases originating from animal reservoirs (Jones et al., 2008). These transmission events arise at complex ecological, biological, and behavioral interfaces that enable pathogens to cross species barriers. Numerous high-consequence spillovers in recent decades underscore the persistent threat posed by wildlife reservoirs and the intricate dynamics of cross-species transmission (Plowright et al., 2017).

NiV, first identified during the 1998 Malaysia outbreak, illustrates the multifactorial nature of spillover. Pteropid fruit bats are the primary natural reservoir, maintaining infection without overt disease (Halpin et al., 2011). Molecular and serologic surveys show NiV circulation across the range of pteropids in Southeast Asia and the Indian subcontinent, establishing broad potential for human exposure (Wacharapluesadee et al., 2021). In Malaysia, a bat to pig to human transmission chain was documented: bats shed virus onto fruit near pig farms, pigs ingested contaminated material and served as amplifying hosts, and humans were infected through close contact with infected pigs (Chua et al., 2000). That outbreak caused 265 human encephalitis cases and 105 deaths and required culling more than one million pigs. Subsequent outbreaks in Bangladesh and India revealed a direct bat to human route through contamination of raw date palm sap, with recurrent outbreaks and case fatality exceeding 70% in some events (Islam et al., 2016). Human-to-human transmission, especially in households and health care settings, has amplified several outbreaks (Gurley et al., 2007).

EBOV disease (EVD) is highly virulent, with case fatality ranging from 25 to 90%. Although non-human primates were initially suspected as reservoirs, current evidence supports fruit bats as the most likely maintenance hosts, while primates act as susceptible intermediate or amplifier hosts (Leroy et al., 2005). The 2013 to 2016 West African epidemic originated from a single spillover in Guinea and resulted in more than 28,600 cases and 11,325 deaths (Holmes et al., 2016). Phylogenetic analyses implicate exposure to insectivorous bats for the index case; subsequent spread was driven primarily by human-to-human transmission via contact with infectious body fluids. Spillover risk is closely linked to forest fragmentation and bushmeat practices. Deforestation increases human bat contact, and hunting, butchering, and consumption of wildlife expose people to tissues with high viral loads (Olivero et al., 2017). Seasonal and environmental factors may also modulate viral shedding in reservoir populations.

Hantaviruses are maintained in specific rodent species with strong host specificity and co-evolutionary patterns, producing distinct viral lineages associated with particular rodent taxa (Jonsson et al., 2010). Human infection typically follows inhalation of aerosolized rodent excreta. Reservoir hosts sustain persistent, generally asymptomatic infections with prolonged shedding (Meyer and Schmaljohn, 2000). The 1993 Four Corners outbreak revealed HCPS in the United States after El Niño associated precipitation expanded vegetation, amplified deer mouse populations, and increased human exposure to Sin Nombre virus (Yates et al., 2002). Similar ecology is observed elsewhere. In Europe, fluctuations in bank vole abundance correlate with Puumala virus outbreaks, and in China, striped field mouse density predicts Hantaan virus incidence (Tian et al., 2017). Agricultural expansion, forest management, and rural poverty modify human rodent interfaces and shape spillover risk.

In addition to these examples, severe acute respiratory syndrome coronavirus-1 (SARS-CoV-1), Middle East respiratory syndrome (MERS-CoV), and SARS-CoV-2 emerged from bat reservoirs, with civets and dromedary camels acting as intermediate hosts for SARS-CoV-1 and MERS-CoV, respectively (Hu et al., 2021). These coronaviruses vary in human-to-human transmissibility, with SARS-CoV-2 achieving exceptional global spread. Common drivers recur across events: wildlife trade and markets concentrate diverse species in close quarters, facilitating cross-species transmission and potential recombination (Wang and Anderson, 2019); agricultural intensification creates amplification interfaces, as seen with NiV in Malaysia and avian influenza in poultry systems. Targeted surveillance at human animal interfaces has detected numerous potential threats before widespread human transmission, including SARS-like bat coronaviruses and novel NiV-like, filo-, and arenaviruses in wildlife reservoirs (Letko et al., 2020). These findings highlight both the vast viral diversity in nature and the feasibility of proactive detection.

### 5.2 Impact of climate change and urbanization

Climate change and urbanization are powerful, intersecting anthropogenic forces that reshape pathogen transmission dynamics and spillover risk on a global scale. These processes modify vector distributions, host-pathogen interactions, and human exposure patterns, creating novel ecological conditions that favor disease emergence.

Arthropod vectors, particularly mosquitoes, ticks, and biting flies, are highly sensitive to climatic variables that determine their geographic distribution. Among these, Aedes mosquitoes (primarily *A. aegypti* and *A. albopictus*) have expanded their ranges with changing climatic conditions, with major implications for arboviral disease distribution (Ryan et al., 2019). *Aedes albopictus*, the Asian tiger mosquito, exemplifies climate-driven range expansion. Originally native to tropical and subtropical Asia, this competent vector for DENV, CHIKV, and ZIKV has established populations across Europe, the Americas, and parts of Africa in recent decades. Statistical models indicate that warming temperatures have made previously inhospitable regions suitable for year-round persistence, with winter isotherm shifts permitting egg survival where conditions were formerly lethal (Kraemer et al., 2019). Projections suggest further expansion under multiple climate scenarios, with estimates indicating that by 2050, approximately 49.5% of the global population may live in zones suitable for year-round *A. aegypti* establishment, an increase of nearly 600 million people compared with current distributions (Liu-Helmersson et al., 2016). Beyond range shifts, higher temperatures can accelerate mosquito development, shorten gonotrophic cycles, and reduce the extrinsic incubation period (EIP) for many arboviruses, increasing vectorial capacity, the mathematical expression of transmission efficiency, even where vectors are already established (Mordecai et al., 2017).

Changes in precipitation patterns further modulate vector ecology in complex and often nonlinear ways. Increased rainfall can generate abundant larval habitats, particularly for container-breeding species such as Aedes and Culex, thereby amplifying vector populations. Conversely, drought conditions may paradoxically elevate transmission risk by promoting water storage practices in urban and peri-urban settings, creating artificial breeding sites in domestic environments. Flooding events can displace both wildlife and human populations, increasing vector-host contact rates and facilitating spillover, while also disrupting sanitation infrastructure and enhancing exposure to vector habitats. Importantly, the relationship between precipitation and transmission is highly context dependent, shaped by local infrastructure, land use, and vector species ecology (Mordecai et al., 2017; Nakhaie et al., 2026).

Extreme weather events including heatwaves, cyclones, and floods, further intensify these dynamics. Heatwaves can transiently increase transmission potential by accelerating viral replication and vector biting rates, although extreme thermal thresholds may also reduce vector survival (Meher et al., 2025). Cyclones and floods disrupt ecosystems and displace reservoir hosts, often forcing wildlife into closer proximity with human populations. Simultaneously, damage to infrastructure can impair vector control programs and limit public health response capacity. Post-disaster conditions, characterized by standing water, population displacement, and weakened healthcare systems, frequently create ideal conditions for rapid outbreak amplification, as documented in multiple arboviral and water-associated disease outbreaks following extreme climatic events (Alqassim, 2025).

Urbanization intersects with climate effects in ways that elevate vector-borne disease risk. The unprecedented pace of global urban growth, with an estimated 68% of the world’s population projected to live in urban areas by 2050, creates ideal conditions for anthropophilic vectors such as *A. aegypti* [United Nations Department of Economic and Social Affairs (UN DESA), 2019; Baker et al., 2022; Rose et al., 2020; Maffey et al., 2022]. Cities provide abundant artificial container habitats for breeding, dense and reliable human blood meals, and sheltered microhabitats that buffer temperature fluctuations. These conditions have driven the urbanization of formerly sylvatic transmission cycles for DENV, CHIKV, and ZIKV. The urban heat island effect, in which cities remain warmer than surrounding rural areas, can sustain *A. albopictus* populations even where regional climate appears unsuitable, creating urban transmission foci ahead of broader regional suitability (Medlock and Leach, 2015). Socioeconomic gradients within cities further structure risk. Informal settlements with intermittent water supply, suboptimal waste management, and limited access to protective measures often experience higher vector densities and greater exposure (Kikuti et al., 2015).

**Figure 3:**
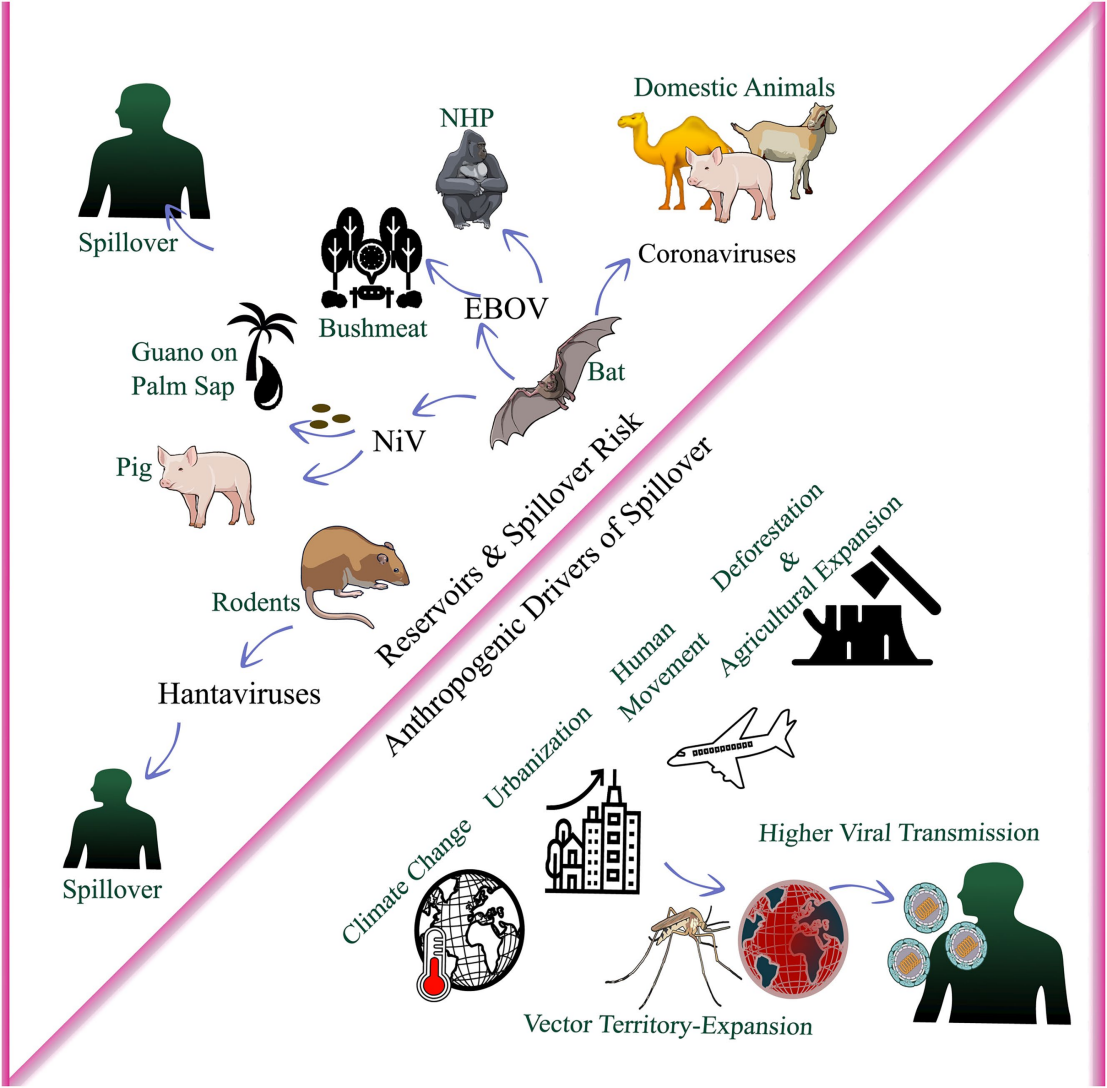
Wildlife reservoirs and anthropogenic drivers of spillover. The figure summarizes how wildlife reservoirs (e.g., bats and rodents) and human-mediated factors (e.g., deforestation and agricultural expansion, urbanization, climate change, and human movement) increase human–animal/vector contact, enabling viral spillover to human populations through pathways such as contaminated food products, bushmeat exposure, and domestic-animal interfaces. NiV, Nipah virus; EBOV, Ebola virus; CoVs, coronaviruses; NHP, non-human primates.

Beyond direct impacts on vector distribution, climate change and urbanization restructure ecological communities and human–wildlife interfaces relevant to spillover. Deforestation, agricultural expansion, and extractive industries extend human activity into previously remote ecosystems, opening pathways for sylvatic pathogens (Gottdenker et al., 2014). Forest fragmentation alters microclimates, can favor certain vectors, and may concentrate wildlife, with evidence from the Brazilian Amazon showing higher arbovirus seroprevalence in mammals from fragmented compared with continuous forests (Moreira-Soto et al., 2018). Climate-driven habitat modification also shifts reservoir host distributions and abundance. Rodent population irruptions after increased precipitation have preceded hantavirus outbreaks globally, while fruit bat range shifts linked to flowering and fruiting phenology may reposition henipavirus risk (Patz et al., 2004). The combined effects of these drivers create ecological conditions without historical precedent, which challenge prediction based on past patterns. Integrated surveillance across human, animal, and environmental health domains is essential for early detection of emerging and re-emerging threats (Kelly et al., 2017). Figure 3 provides a visual summary of the discussed reservoir and anthropogenic drivers of viral spillover and transmission.

## 6 Computational models and pandemic prediction

### 6.1 Mathematical foundations of epidemiological modeling

Mathematical models have a long history in epidemiological research, and compartmental models act as fundamental tools to understand disease transmission dynamics (Aguiar et al., 2016). Depending on the epidemiological status, these models divide a population into distinct compartments such as susceptible, exposed, infectious, and recovered (SEIR). R0 denotes the mean number of secondary infections from an infectious case in a fully susceptible population, acting as the critical threshold parameter; R0 > 1 indicates epidemic potential (Diekmann et al., 1990). For vector-borne diseases, mathematical models require considering the population dynamics of both the host and the vector simultaneously. The Ross–Macdonald model, considered the cornerstone of malaria epidemiology, demonstrates the association between R0, vector density, biting rate, and the rate of pathogen development (Ross, 1911). In modern extensions, SEIR models incorporate R₀ = *β*/*γ*, where β denotes transmission rate and γ the recovery rate. From a biological and public health standpoint, this relationship implies that reducing β through vector control, personal protection, or social distancing, or increasing γ through effective treatment, can drive R₀ below the critical threshold of 1 and thereby ensure epidemic extinction (Wearing et al., 2005). This interpretation bridges the mathematical formulation to actionable intervention strategies, a consideration central to the practical utility of these models.

It is important to note that while mathematical models offer valuable insights into disease transmission, their interpretation requires careful consideration of underlying factors. Parameters like vector biting rates, mosquito survival, and the EIP are significantly shaped by environmental conditions including temperature, humidity, and the presence of urban infrastructure. Therefore, model predictions should be viewed as approximations rather than definitive forecasts. Future modeling initiatives should, therefore, emphasize collaboration across disciplines including epidemiology, climate science, and data science to enhance our ability to prepare for and respond to outbreaks effectively (Vynnycky and White, 2010; Drake et al., 2021; Gog et al., 2015).

### 6.2 Compartmental models for vector-borne diseases

#### 6.2.1 Applications of SIR and SEIR models

A comparative study on SIR, SEIR, and SIR-SI models in Panama shows that all three models estimated R0 to range from 1.09 to 1.74 in the context of dengue. This is considered the first calculation of R0 for a dengue outbreak in Panama. In a comparative performance analysis, the SIR model had a strong tendency to estimate the effective susceptible population, while the SIR-SI model estimated lower population figures. The SEIR model predicted a population size between that of the other two models (Valencia et al., 2023). An advanced SEIR-SEI model for dengue was developed by considering the psychological effects such as anxiety and stress (Hasan et al., 2022). The analysis of susceptibility showed that the natural mortality rate (*μ*₂) of the vector population is the most important susceptible parameter of the system. Using the artificial neural networks (ANNs) along with particle swarm optimization (PSO) with local search algorithm, the SEIR model was solved for ZIKV with an absolute error ranging from 10^−12^ to 10^−6^ (Sabir et al., 2023).

#### 6.2.2 Agent-based models as a complementary approach

Agent-based models (ABMs) offer a complementary framework to classical compartmental approaches by simulating individual human and vector agents within spatiotemporal environments, rather than treating populations as homogeneous groups. Unlike SIR-type models that aggregate population dynamics into differential equations, ABMs can explicitly represent heterogeneous host-vector interactions, fine-scale human mobility, and the full mosquito life cycle. For instance, Mahmood et al. proposed an ABM framework incorporating a dedicated bite algorithm alongside *Aedes* mosquito life-cycle stages and environmental temperature data to predict dengue case distribution and the directionality of spread, a capability with direct relevance to early outbreak detection and emergency preparedness (Mahmood et al., 2020).

#### 6.2.3 Multiple-serotype models

For diseases like dengue that have multiple serotypes, complex models have been developed to consider the effects of ADE. Ferguson et al. developed a multiple-strain model that investigated the effect of ADE on the transmission dynamics of dengue. Their model showed the deterministic chaotic dynamics under the assumption of a subsequent severe infection by DENV (Ferguson et al., 1999). Subsequent incorporation of temporary cross-immunity (TCI) periods revealed a substantially wider parameter window for complex dynamics, highlighting that the interplay between ADE and TCI, rather than the exact number of serotypes, is the dominant driver of long-term epidemiological complexity (Aguiar et al., 2008).

### 6.3 Climate-dependent models

#### 6.3.1 Temperature-dependent parameters

Transmission of vector-borne diseases indicates the strong temperature-dependence through various pathways. Temperature affects the vector survival, development rate, biting behavior, and pathogen replication within the vector. R0 for vector-borne diseases includes these temperature-dependences:


$$R^{0}=\sqrt{\frac{\left(p_{\mathrm{hv}(T)}\cdot B(T)\cdot e^{-d(T)\cdot \tau_{v(T)}}\cdot U*(T)\right)}{d(T)\cdot H*}}$$

Where T is temperature, p_hv(T) is host-to-vector transmission probability, B(T) is biting rate, d(T) is vector mortality rate, and τ_v(T) is the EIP. The public health implication is direct: as temperature deviates from the optimal transmission range, the multiplicative effect of these parameters substantially reduces R₀, providing a biological rationale for seasonally targeted control efforts. Research has confirmed that ZIKV is transmitted by *Aedes aegypti* at an optimal temperature of 29 °C, within a viable range of 22.7–34.7 °C (Tunali et al., 2021). Projections for ZIKV in Brazil under the RCP4.5 climate scenario indicate peak incidence rising from 10,473 to 22,030 cases during 2070–2,100, underscoring the urgency of integrating climate projections into disease surveillance systems, a need documented across multiple early warning frameworks (Mahmood et al., 2020; Ferguson et al., 1999; Aguiar et al., 2008).

#### 6.3.2 Predictive climate indices

The Mosquito-borne Viral Suitability index (index P) was developed to estimate transmission risk for DENV, CHIKV, and ZIKV (Carreto et al., 2022). Validated against dengue incidence data in Mexico, the index showed city-level correlations ranging from 0.25 (Cancún) to 0.86 (Campeche), reflecting substantial geographic heterogeneity in its predictive power, a finding consistent with evidence that vector-borne disease prediction at national or continental scales is less reliable than locally calibrated models, given regional variation in climate, vector ecology, and disease dynamics (Kiryluk et al., 2024).

### 6.4 Machine learning approaches

Seasonal autoregressive integrated moving average (SARIMA) models were used for predicting the transmission pattern of CHIKV infection in Brazil using data from 2017–2019. They showed how Chikungunya transmission has transitioned, making it more predictable rather than sporadic and explosive. SARIMA models predicted 106,162 serologically confirmed cases and 339,907 total notifications for 2022–2023 (Yakob, 2022).

Hybrid deep learning models, such as convolutional neural network–long short-term memory (CNN-LSTM) and generative adversarial network-gated recurrent units (GAN-GRU) outperformed the basic machine learning models. The CNN-LSTM model achieved the best performance with an averaged absolute percentage error of 3.718% (Dairi et al., 2021). The machine learning-based models applied using superficial musculoaponeurotic system (SMAS) method led to the identification of IFI6 and IFI27 in the context of NiV, indicating the prediction performance with an accuracy of 100% (Rezapour et al., 2024).

Research studies on 49 early warning systems showed that machine learning models, such as Gated Recurrent Unit, Random Forest, and Boosted Regression Tree have been used for detection and prediction of infectious diseases. Social media-based early warning systems are capable of predicting the incidence of Coronavirus disease 2019 (COVID-19) infection 6–27 days earlier than official reports, a finding with direct implications for real-time public health response (Li et al., 2024).

The study by Reich et al. on 490,210-point prediction showed that the non-mechanical and ensemble models outperformed the compartmental models in short-term, however, the orders reversed beyond 4–5 weeks of projection horizon. This pattern reflects a fundamental trade-off: data-driven models excel at capturing recent trends without mechanistic assumptions, whereas structural models preserve biological plausibility over longer timescales. Research studies showed that the models failed when they used more speculation and theoretical assumptions and tried to predict long-term outcome (Rahmandad et al., 2022; Xiang et al., 2021).

One of the main problems is associated with the input data quality. The daily new confirmed cases number does not reflect the actual number of new infections on that date due to limited tests and recommendations from health organizations. For models that use exponential variables, small errors can lead to large deviations from reality (Petropoulos et al., 2022).

Including the combination of physical/mechanical modeling and machine learning techniques with consideration of human behavioral dynamics and resetting state variables. The future of the modeling also lies in the development of the AAAR framework that integrates real-time data with machine learning algorithms, natural language processing (NLP), and real-time data streams from different sources (Rahmandad et al., 2022; Lou et al., 2024).

Real-time diagnostic devices, including point-of-care diagnostic tools, wearable sensors, and remote monitoring systems have been integrated with data integration platforms, such as Apache Kafka and Apache Flink, to process real-time data. These advances allow for the development of early warning systems that alert health authorities about the emerging outbreaks (Udegbe et al., 2023). Computational models for vector-borne and other zoonotic RNA viruses are rapidly evolving and provide powerful tools for perception, prediction and control of these diseases. The continuation of climate change, urbanization, and global connectivity, which changes the disease transmission patterns, makes essential the use of complex modeling approaches to protect global health security.

From a public health standpoint, computational modeling plays a crucial role in early warning systems. Forecasting models can help pinpoint regions facing a heightened risk of outbreaks, inform targeted vector-control strategies, and support the efficient allocation of healthcare resources (Chowell and Skums, 2024). However, the accuracy of these predictions is strongly reliant on the quality of available data, the extent of surveillance efforts, and the incorporation of factors like human behavior and environmental conditions (Keshavamurthy et al., 2025; Chowell and Skums, 2024).

## 7 Novel strategies for prevention and control

### 7.1 Vector control strategies

The emergence and resurgence of arboviruses have accelerated over recent decades due to climate change, rapid urban development, and globalization, which together facilitate the spread of blood-feeding arthropods into new regions. Arthropod vectors include mosquitoes, ticks, fleas, sandflies, and other species capable of transmitting pathogens between hosts. Many vectors tolerate high levels of viral replication with minimal pathology, enabling efficient transmission (Socha et al., 2022). Early recognition of arthropods as vectors for viral disease led to large-scale prevention campaigns, beginning with YFV control efforts in the Americas in the early twentieth century. Contemporary strategies for preventing and managing arboviral transmission fall into two broad categories: interventions targeting vectors and interventions targeting the viruses themselves, including vaccines and therapeutics (Socha et al., 2022). Vector-focused approaches aim to reduce local vector abundance, limit contact with human hosts, or diminish vector competence. Core methods include mechanical, chemical, and biological control (Socha et al., 2022; Ejaz et al., 2024).

#### 7.1.1 Mechanical control

Mechanical measures are long-standing, cost-effective components of vector control for mosquitoes and ticks. Key practices include eliminating or covering containers that hold standing water, which serve as primary mosquito breeding sites; maintaining clean streets, buildings, and residential areas; and using personal protective measures such as long-sleeved clothing and topical repellents. Installing and maintaining window and door screens reduces indoor mosquito entry and human exposure. In tick-endemic areas, careful inspection of clothing, luggage, and pets after outdoor activities reduces tick bites and pathogen exposure (Ejaz et al., 2024). Vector surveillance and suppression can be augmented with traps that attract and capture mosquitoes at scale (Socha et al., 2022). Mass-trapping programs have maintained *Aedes aegypti* populations at low levels for extended periods, and communities where these interventions were deployed have shown lower serologic evidence of arboviral exposure. Recent advances in trap design and deployment show promising results in laboratory and small field trials, including auto-dissemination strategies in which mosquitoes transfer control agents to breeding sites. Larger, rigorously designed field studies with both entomological and epidemiological endpoints are needed to define effectiveness and operational best practices (Barrera, 2022).

#### 7.1.2 Chemical control

Chemical control has been central to vector management since the introduction of dichloro-diphenyl-trichloroethane (DDT) in the 1940s. Intensive residual spraying during the 1950s and 1960s, combined with larval source reduction, contributed to regional *Aedes aegypti* suppression in South America and reduced YFV and DENV transmission. Today, chemical interventions focus on larvicides and adulticides. Larvicides include insect growth regulators (IGRs) such as pyriproxyfen, methoprene, and diflubenzuron (Socha et al., 2022). Field studies report that IGRs, including pyriproxyfen, can effectively reduce immature Aedes populations (Unlu et al., 2017). In many settings, larval control is prioritized for *Aedes aegypti* using chemical or microbial larvicides in combination with IGRs (Ejaz et al., 2024). Continued reliance on insecticides raises concerns about human exposure, ecological effects, and the evolution of insecticide resistance, underscoring the need for resistance management and integrated vector management frameworks (Socha et al., 2022).

However, the long-term effectiveness of chemical vector control strategies is increasingly compromised by the widespread emergence of insecticide resistance, particularly to pyrethroids, in major vector species such as *Aedes aegypti* and *Aedes albopictus* (Godson, 2026). Resistance mechanisms, including target-site mutations and metabolic detoxification, have been reported across multiple endemic regions, reducing the efficacy of conventional interventions. In addition, the environmental persistence and non-target effects of chemical insecticides raise ecological and public health concerns, further limiting their sustainability. These challenges have prompted a shift toward integrated vector management approaches that combine chemical, biological, and environmental strategies to achieve more durable control outcomes (Samal et al., 2022; Tiffin et al., 2025).

#### 7.1.3 Biological control

Biological control leverages natural enemies of vectors—predators, parasites, or pathogens—to suppress populations or reduce transmission. Approaches include entomopathogenic fungi, augmentation of natural predators, and the use of bacterial larvicides as biological toxins (Socha et al., 2022). A particularly promising strategy is the use of Wolbachia, an intracellular endosymbiont present in more than 70% of insect species. Wolbachia colonizes the gonads and is maternally transmitted. Although *Aedes aegypti* does not naturally harbor Wolbachia, stable trans-infections can be established by microinjection of strains sourced from hosts such as *Aedes albopictus* or *Drosophila melanogaster* (Socha et al., 2022). Wolbachia infection can impose fitness costs on mosquitoes, including reduced adult longevity in some strain–host combinations (Ogunlade et al., 2021). It can also suppress arbovirus transmission through several mechanisms: cytoplasmic incompatibility that reduces viable offspring when infected males mate with uninfected females; potential lifespan shortening that limits the time available for transmission; and direct “pathogen blocking,” in which specific Wolbachia strains reduce replication of viruses such as DENV, ZIKV, and CHIKV within the mosquito (Socha et al., 2022; Ejaz et al., 2024; Pinto et al., 2021). Field releases of Wolbachia-infected *Aedes aegypti* in Indonesia have been associated with substantial reductions in dengue incidence (Indriani et al., 2020). Population-suppression strategies based on releasing only infected males require accurate sex sorting, which increases operational complexity and cost. Viral evolutionary escape from Wolbachia-mediated blocking is considered possible but likely constrained because it would require multiple coordinated mutations that preserve fitness in both human and mosquito hosts (Socha et al., 2022).

### 7.2 Vaccines and antiviral treatments

Vaccination remains one of the most effective tools for preventing infectious diseases, but only a limited number of licensed vaccines exist for major arboviruses. The live-attenuated 17D YFV vaccine has been in use since the 1930s, with current products derived from 17D sub-strains and only modest compositional changes since the 1980s (Socha et al., 2022; Ejaz et al., 2024). Despite durable protection, recognized limitations include rare neurotropic and viscerotropic adverse events, egg-protein hypersensitivity in some recipients, and labor-intensive production. Novel YF vaccine candidates include inactivated formulations generated with beta-propiolactone or hydrogen peroxide, subunit approaches expressing pre-membrane and envelope (prM/E) proteins, and DNA constructs encoding YF envelope (Socha et al., 2022). Vaccine development for DENV is complicated by four antigenically distinct serotypes and the risk of ADE. The only licensed product to date is a live-attenuated, chimeric YFV 17D-based tetravalent vaccine (Dengvaxia^®^), in which prM/E sequences from each DENV serotype replace the corresponding YF sequences. Protection can persist for several years, but effectiveness varies by serotype, age, and pre-vaccination serostatus (Socha et al., 2022). For JEV, four vaccine platforms have been developed. The mouse brain-derived inactivated vaccine (JE-VAX^®^) was immunogenic but posed safety and manufacturing challenges and is no longer in use in many countries. Cell-culture–derived inactivated vaccines are now widely used and offer a favorable safety profile. A live-attenuated cell-culture vaccine in use in China provides high protection but carries a theoretical risk of reversion. A live-attenuated chimeric vaccine based on the YF-17D backbone incorporating JEV prM/E sequences has also been developed (Socha et al., 2022).

Recent advances in vaccine development platforms have accelerated efforts against several emerging arboviruses and zoonotic RNA viruses. In particular, nucleic acid–based vaccines, including mRNA technologies, have demonstrated rapid scalability and adaptability, as highlighted during the COVID-19 pandemic, and are now being explored for pathogens such as ZIKV, DENV, and CHIKV. Similarly, virus-like particle (VLP)-based vaccines offer advantages in safety and immunogenicity due to their ability to mimic native viral structures without containing infectious genetic material. In parallel, novel antigen display systems such as protein nanocages and self-assembling nanoparticles are being investigated to enhance immunogenicity and enable multivalent vaccine design. Despite these advances, most candidates remain in early-stage development, and challenges related to durability of immunity, manufacturing scalability, and equitable access continue to limit their near-term impact in endemic regions (Ormundo et al., 2023; Pujhari, 2023).

RNAi is being explored primarily as a vector-control modality, with studies targeting essential mosquito genes involved in survival, reproduction, and behavior in species such as *Aedes aegypti*, *Anopheles gambiae*, *Culex quinquefasciatus*, and Aedes subalbatus. Delivery of complementary RNA molecules can disrupt key pathways and suppress vector fitness (Agarwal et al., 2022).

Despite its promise, RNAi-based vector control remains largely at the proof-of-concept stage, with most studies confined to laboratory or semi-field settings. Key challenges include the efficient delivery of dsRNA in natural environments, stability under field conditions, and scalability for large-scale implementation. While recent advances in nanoparticle-mediated delivery and transgenic approaches have improved RNAi efficiency in mosquitoes, these strategies have not yet been translated into operational vector control programs. Therefore, RNAi should currently be considered an experimental tool with potential future applications rather than a readily deployable intervention (Maya-Maldonado et al., 2025).

Immunotherapy with monoclonal antibodies (mAbs) provides a complementary strategy for prevention and treatment. Neutralizing mAbs have been discovered against DENV, ZIKV, CHIKV, WNV, and tick-borne encephalitis virus (TBEV) using modern antibody-discovery and engineering platforms. mAbs can also guide vaccine antigen design by revealing protective epitopes and mechanisms of neutralization. Key challenges include achieving potency, breadth across diverse viral genotypes or serotypes, and durable protection, while minimizing enhancement risks for flaviviruses. Protein engineering and optimized Fc effector functions are active areas of development to overcome these barriers (Ormundo et al., 2023).

**Figure 4:**
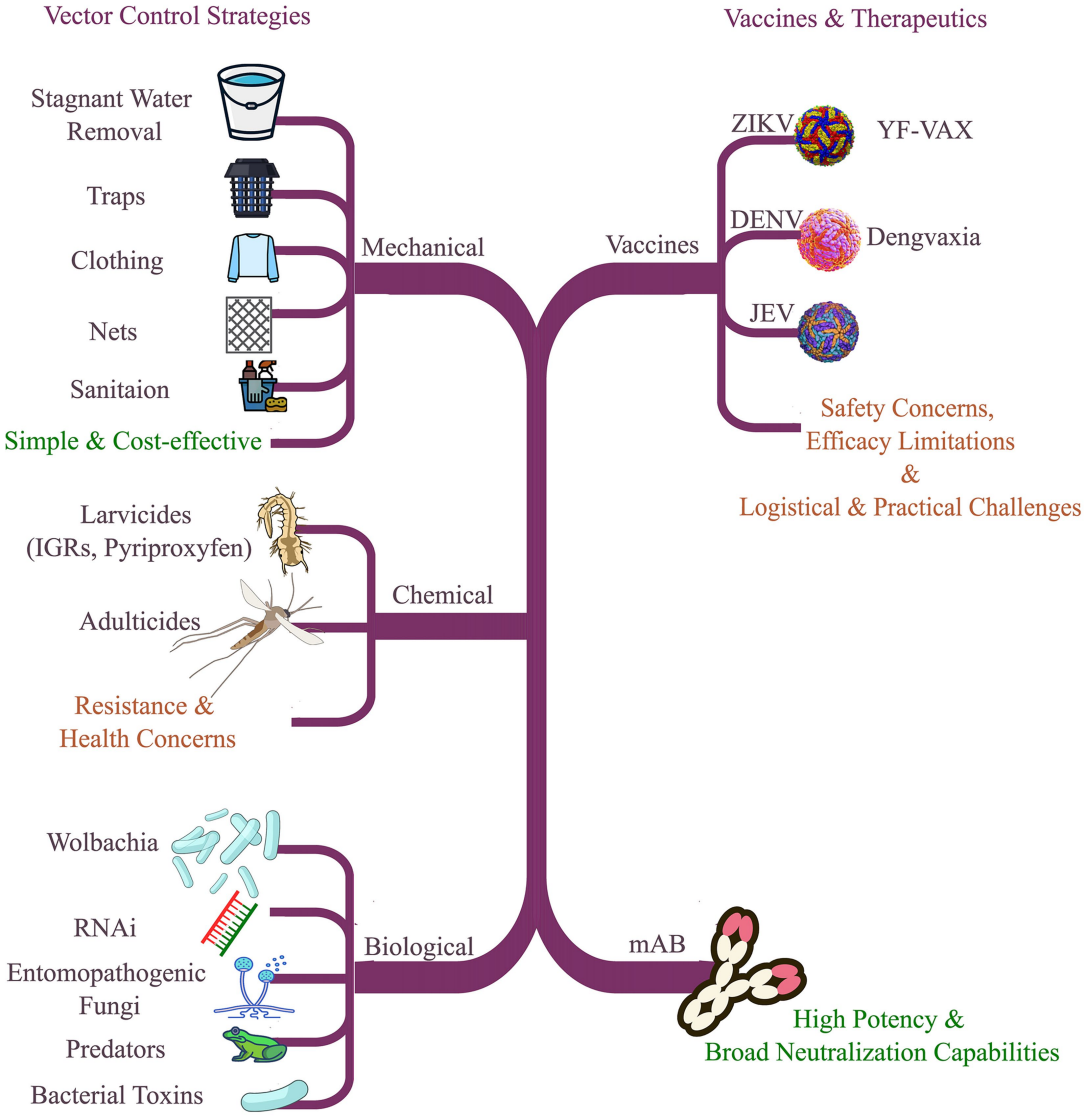
Summary of vector control, vaccination, and therapeutic approaches for prevention and control of vector-borne and other zoonotic viral diseases. Vector control strategies (source reduction, sanitation and stagnant-water removal; personal protection such as clothing and nets; and mechanical, chemical, and biological approaches including traps, larvicides, adulticides, predators, bacterial toxins, entomopathogenic fungi, *Wolbachia*, and RNAi) with vaccines and therapeutics (licensed or available vaccines such as YF-VAX and vaccines for DENV, ZIKV, and JEV, as well as antibody-based therapies [mAbs]). Key implementation considerations highlighted include simplicity, cost-effectiveness, insecticide resistance and health concerns, and safety, efficacy and logistical limitations for vaccines and therapeutics. DENV, dengue virus; ZIKV, Zika virus; JEV, Japanese encephalitis virus; YF-VAX, yellow fever vaccine; IGRs, insect growth regulators; mAbs, monoclonal antibodies; RNAi, RNA interference; *Wolbachia*, *Wolbachia* endosymbiont–based vector control.

Overall, sustained progress will depend on integrated vector management that combines environmental, biological, and chemical tools; targeted vaccination where available; and new antivirals and immunotherapies (Figure 4). Strategic deployment, rigorous field evaluation, and resistance management will be essential to ensure impact and durability.

## 8 Conclusion

The continuing expansion of vector-borne and other zoonotic RNA viruses underscores the delicate balance between humans, animals, and the environment. As climate variability, globalization, and urban crowding intensify, these infections are expected to become more frequent and unpredictable. Strengthening surveillance systems, improving diagnostic and modeling capacities, and encouraging collaboration between public health, veterinary, and environmental sectors are critical steps toward early detection and control. The integration of advanced computational tools with traditional epidemiology can transform how we anticipate and respond to outbreaks. Ultimately, sustainable prevention will rely on global cooperation, investment in research, and policies that address the environmental and social determinants of disease emergence.
