# Correction: Emerging and re-emerging vector-borne and other zoonotic RNA viruses: pathogenesis, climate-driven dynamics, and strategies for global control

**DOI:** 10.3389/fmicb.2026.1882311

**Published:** 2026-05-28

**Authors:** Frontiers Production Office

**Affiliations:** Frontiers Media SA, Lausanne, Switzerland

**Keywords:** climate change, global health, pathogenesis, vector-borne viruses, zoonotic diseases

The order of authors in the author list of the published paper was erroneously given as:

“Niloofar Farsiu, Mohammad Rezaie Zadeh Rukerd, Fatemeh Khodadadpour Mahani, Nazanin Zeinali Nezhad, Mohammad Rahimi, Fatemeh Sadat Mirtajadini, Mohammad Pardeshenas, Mohsen Nakhaie and Pouya Hassandarvish”.

The correct author list reads:

“Niloofar Farsiu, Fatemeh Khodadadpour Mahani, Mohammad Rezaie Zadeh Rukerd, Nazanin Zeinali Nezhad, Mohammad Rahimi, Fatemeh Sadat Mirtajadini, Mohammad Pardeshenas, Mohsen Nakhaie, Pouya Hassandarvish”.

The original version of this article has been updated.

